# Reliability and Validity of the East Asia-Pacific Early Child Development Scales: A Longitudinal Validation Study in China

**DOI:** 10.1007/s10578-023-01526-9

**Published:** 2023-03-29

**Authors:** Nirmala Rao, Yufen Su, Stephanie W. Y. Chan

**Affiliations:** Faculty of Education, The University of Hong Kong, Pokfulam Road Hong Kong, Hong Kong SAR, China

**Keywords:** EAP-ECDS, Early child development, Reliability, Validity

## Abstract

This study examined the test-retest reliability and predictive validity of the East Asia-Pacific Early Child Development Scales (EAP-ECDS) Short Form. In China, preschools typically provide children with educational activities in age-segregated classrooms – Kindergarten Level 1 (K1) (3 to 4 years), Kindergarten Level 2 (K2) (4 to 5 years), and Kindergarten Level 3 (K3) (5 to 6 years). A total of 709 children in K2 (*M*_age_ = 57.85 months, *SD* = 4.77) were randomly selected from 29 kindergartens in Shanghai municipality and Guizhou province of China. Children were assessed using the EAP-ECDS in K2 and K3. School readiness was assessed in K3, and literacy and mathematics achievement were assessed in Grade 2. Pearson’s correlation coefficient and intraclass correlation coefficient (*ICC* = 0.73) indicated that the tool had good test-retest reliability across K2 and K3. Regarding predictive validity, K2 EAP-ECDS predicted K3 school readiness (β = 0.26), Grade 2 language and literacy (β = 0.18) and mathematics (β = 0.22) after adjusting for age, gender, socioeconomic status, and region. Findings support using the tool to measure the holistic development of preschool-aged children in China and the region.

The knowledge, capacities, and skills developed in the first five years of life form a strong foundation for children’s later learning and development [1, 2]. The significance of early childhood development (ECD) is reflected in Sustainable Development Goals (SDG) Target 4.2, which states that all children should have access to quality early education services to be ready for primary education [3]. SDG Target Indicator 4.2.1 is the proportion of children aged 24–59 months who are developmentally on track in health, learning, and psychosocial well-being. It is critical to have psychometrically robust tools to track progress toward Target Indicator 4.2.1 in low- and middle-income countries, where many young children are not reaching their developmental potential [1].

It is generally acknowledged that ECD measurements should capture children’s competencies across multiple interdependent developmental domains [4–6]. Traditionally, developmental screening tools such as the Denver Developmental Screening Test have been widely used among pediatricians to assess individual children’s early development [4]. However, screening tools lack the sensitivity to detect changes in ECD caused by educational services or policy changes at scale [7]. In recent years, several psychometrically robust tools have been developed for population-based monitoring of ECD, such as the UNICEF’s Early Childhood Development Index (ECDI), the Early Human Capability Index (eHCI) [6] and the Early Childhood Development Assessment Scale (ECDAS) [5]. With increasing access to early childhood education in middle-income countries, particularly in China [8], having psychometrically robust tools to evaluate the efficacy of educational programs and identify young children’s strengths and difficulties in learning and development are also essential. The International Development and Early Learning Assessment (IDELA) is one such tool [9]. It has been used in about 80 countries and is recommended for monitoring and program evaluation within a country rather than for comparisons across countries [10]. Another tool used for measuring child development and program evaluation is the East Asia-Pacific Early Child Development Scales (EAP-ECDS) [11].

The EAP-ECDS [11] is a culturally and contextually sensitive direct assessment tool developed to assess three- to five-year-old children’s early development. The tool was developed based on the early learning and development standards of six East Asia-Pacific countries and assesses seven interdependent domains of ECD, namely Cognitive Development, Language and Emergent Literacy, Social-emotional Development, Motor Development, Health, Hygiene and Safety, Cultural Knowledge and Participation, and Approaches to Learning. The tool has been validated with representative samples in the region in seven countries (i.e., Cambodia, China, Mongolia, Myanmar, Papua New Guinea, Timor-Leste, and Vanuatu). Results indicated good psychometric properties of the measurement regarding content validity and internal consistency. Previous analyses showed strong correlations between child age, gender, family socioeconomic status and child’s performance on the tasks. This indicates that the measurement was sufficient to differentiate children’s competencies based on their sociodemographic characteristics [11–13]. In 2016, the tool was shortened to decrease administration time and improve its feasibility for population use [14].

The original and short-form versions of the EAP-ECDS have been used in empirical studies to measure child development and evaluate ECE program effectiveness in the East Asia-Pacific region. For example, studies identified associations between developmental domains with nutritional status and body composition indicators [15, 16], parental engagement [12], socioeconomic status [12], and teacher-reported approaches to learning [17]. Studies have also found positive associations between EAP-ECDS domains with preschool attendance, early interventions, and home learning activities, providing support for the benefits of early childhood education programs and home environments on specific aspects of child development [18–20]. The EAP-ECDS has also been used as the outcome measure of child development to evaluate the criterion validity of measures [21], to examine secular trends over time [22], and to compare associations between direct assessment and adult-reported measures [23]. The original validation study of the scale demonstrated high internal consistency and excellent item discrimination ability across six countries (Cambodia, China, Mongolia, Papua New Guinea, Timor-Leste, and Vanuatu) [11]. With the increased usage of the scale, it is essential to evaluate EAP-ECDS’s psychometric properties in terms of test-retest reliability and predictive validity.

Test-retest reliability concerns the “consistency, reproducibility, and agreement among two or more measurements of the same individual, using the same tool, under the same conditions” [24]. There are two indices of test-retest reliability: relative test-retest reliability (relative consistency) and absolute test-retest reliability (absolute agreement) [24, 25]. Relative test-retest reliability concerns the consistency of children’s positions in a group relative to their peers [25]. Absolute test-retest reliability examines the extent to which the outcomes observed with the measurement tool are the same over time, raters, or context when assessing the same unchanged individuals [24]. Psychologists have been cautious about determining the test-retest reliability of a psychological construct, as the reliability may vary with changes in individuals, memory, desire for consistency, time interval, practice effects, and other factors. Absolute test-retest reliability is particularly sensitive to the retest interval, with the coefficient declining as the interval increases [26].

The goal of the present study was to examine the test-retest reliability and predictive validity of the EAP-ECDS (SF) over four years, involving children from diverse demographic backgrounds in China. Test-retest reliability was examined by computing the association between the EAP-ECDS (SF) score assessed in Kindergarten Level 2 (K2; the second year of preschool education for children aged 4 to 5 years) and the EAP-ECDS (SF) score obtained at the end of Kindergarten Level 3 (K3; the third year of preschool education for children aged 5 to 6 years). Because of the long intervals between the wave and the nature of the skills measured, we examined the relative test-retest reliability of the measurement tool rather than the absolute test-retest reliability that has been more commonly reported in the literature. The EAP-ECDS (SF) is a developmental assessment tool. That said, children were expected to perform better on the measure due to rapid developmental change during the preschool period and preschool experience. In addition, as there was a retest interval of one year and a half, it was unrealistic to expect high absolute agreement coefficients over two time points. Therefore, looking at relative test-retest reliability in this study is more meaningful. Predictive validity was investigated by examining the relation between the EAP-ECDS (SF) and school readiness as evaluated by the Bracken School Readiness Assessment - Third Edition (BSRA-3) [27] assessed in K3 and children’s academic achievements (i.e., language and literacy, mathematics) assessed in Grade 2.

## Method

### Participants

The participants of this study were from a four-year longitudinal study on the effects of preschool quality on children’s later academic achievement. The project took place in Guizhou and Shanghai, two regions that varied substantially in socioeconomic and educational development. Given the significance of the geographic location of household residence in Chinese children’s development, children were chosen from various administrative districts (cities) to maximize sample diversity. In Shanghai, children were selected from kindergartens in 13 administrative districts (5 urban, 7 suburban, and 1 rural). In Guizhou, children were selected from 16 kindergartens in urban areas, townships, county centers, and villages of four cities/regions with diverse socioeconomic development.

The initial sample consisted of 709 children (*M*_age_ = 57.85 months, *SD* = 4.77) randomly selected from 52 K2 classrooms within 29 kindergartens. Children were assessed at three time points: they were followed from the fall semester of K2 (Wave 1) to the end of the spring semester of K3 (Wave 2) and then to the fall semester of Grade 2 (grade level for 7-year-olds; Wave 3). There was sample attrition as the project went on. In K2, 705 children completed the EAP-ECDS (SF). In K3, 538 children (77% of the initial sample) were administered the EAP-ECDS (SF) and the school readiness test. In Grade 2, 316 children (45% of the initial sample) completed the academic tests. We compared children with valid data for all three time points and those who dropped out in K2 and/or K3. The analyses showed that children who dropped out were more likely to be from Shanghai (68%) than Guizhou (32%) and from families with higher parent education (*t* = 4.6, *p* < .001) and household wealth (*t* = 5.06, *p* < .001). Indeed, children from Shanghai had parents with higher education levels and lived in families with higher household wealth than those in Guizhou. Children were excluded from the original sample if they were older than 71 months in K2, their EAP-ECDS (SF) scores were 3 SD lower than the average, and they were over 6 years in K3. As a result, 687 children were included in the final analyses. Table 1 summarizes the demographic characteristics of child participants.

**Table 1:** Sample demographics

	N	Mean (SD)	n/%	Range
Child age (months)	687	57.74 (4.56)		37–71
Child gender (boy)	687		361 (52.55)	
Length of preschool exposure	604	4.47 (1.36)		1–8
Paternal education	635	5.74 (1.47)		1–9
Maternal education	634	5.76 (1.44)		1–9
Household wealth (K2 )	635	0.05 (2.39)		-4.36-8.66
Household income (Grade 2)	211	6.21 (3.04)		1–9
Home learning environments	620	5.09 (3.04)		0–6
Region of household residence	687			
Urban Shanghai			119 (17.32)	
Suburban Shanghai			262 (38.14)	
Urban Guizhou			194 (28.24)	
Rural Guizhou			112 (16.30)	

## Procedure

In K2 and K3, trained assessors directly assessed children in their kindergartens. In Grade 2, constrained by strict school visitor policy during the COVID-19 pandemic, children were administered the academic tests (i.e., language and literacy and mathematics) in their homes. The parents were given instructions on implementing the test and requested to submit scanned copies of the completed tests to the research team. Before data collection, consent was obtained from parents or other caregivers (e.g., grandparents). In K2 and Grade 2, caregivers of children also completed questionnaires tapping sociodemographic information and home learning environments. All sampling methods and procedures described here were reviewed and approved by (blinded for review).

Before data collection, assessors participated in a 3-day training that covered the theoretical basis, constructs, administration, and scoring of the EAP-ECDS (SF). The training also included a real-setting practice wherein all the assessors were given an opportunity to administer the scale to children. Each assessor had to rate at least 85% of the items with identical scores as the supervisor – an EAP-ECDS (SF) expert - before collecting data.

## Measures

### EAP-ECDS (SF)

The 33-item EAP-ECDS (SF) is a derivative of the long version EAP-ECDS developed based on the Early Learning and Development Standards of countries in the East Asia-Pacific region [14]. As described in the introduction, the SF measures seven domains of development, namely Cognitive Development (α_K2_ = 0.90, α_K3_ = 0.83), Social-emotional Development (α_K2_ = 0.8 = 79, α_K3_ = 0.70), Motor Development (α_K2_ = 0.50, α_K3_ = 0.55), Language and Emergent Literacy (α_K2_ = 0.82; α_K3_ = 0.67), Health, Hygiene, and Safety (α_K2_ = 0.68; α_K3_ = 0.47), Cultural Knowledge and Participation (α_K2_ = 0.72; α_K3_ = 0.74), and Approaches to Learning (α_K2_ = 0.81; α_K3_ = 0.84). The EAP-ECDS (SF) took approximately 35 min to administer, and the duration of the session varied depending on child age, ability, mood, and rapport with the assessor.

### School Readiness

Children’s school readiness was assessed with the BSRA. The BSRA is widely used to measure early language and basic concept attainment for children aged from two years six months to seven years eleven months [28]. This study used six subtests of this scale to measure children’s early literacy and mathematics competence: colors, letters, numbers and counting, comparison, and shapes (α = 0.89). The Chinese adaption of these subtests has good reliability and validity in rural China [28].

### Academic Achievement

In 2020 (Grade 2), children completed academic tests of Chinese language and literacy and mathematics. The two tests were developed based on the *National Curriculum Guidelines for Basic Education* (MOE, 2011) and workbooks used by students. Experienced primary school teachers reviewed the items to ensure the difficulty and length were appropriate for Grade 2 children. The language and literacy test (α = 0.93) assessed children’s knowledge in three areas: Chinese characters (e.g., pronunciations, writing, Pinyin, antonyms and synonyms), reading comprehension, and writing. Examples of test items include “*Write the Chinese characters in Pinyin*”, “*Find the antonyms and connect the following words*”, and “*Rearrange the following words to form a sentence and add punctuation*”. The mathematics test (α = 0.91) assessed children’s knowledge of numeracy and geometry. Sample items include “*please identify the patterns and write down the following numbers*” and “*please identify what should be above, below, and on the right and left sides of the bicycle in the picture*”.

### Covariates

We included six covariates that were shown to relate to children’s developmental outcomes and academic achievement in previous studies with the EAP-ECDS [12, 19]. These included child age in months, child gender (boy = 1, girls = 0), and length of preschool attendance. Children’s home learning environments in K2 were also included, measured by a scale comprised of six dichotomous questions (requiring yes or no responses) from the Multiple Indicator Cluster Surveys (α = 0.79) [29]. These questions ask parents (or other primary caregivers who responded to the questionnaire) whether any family members aged above 15 years had provided the following activities at home for the child in the past three days: (i) reading books; (ii) telling stories; (iii) singing songs; (iv) taking children outside the home place; (v) playing with the child; and, (vi) engaging the child in naming things or counting. Total scores ranged from 0 to 6. A family socioeconomic status (SES) measure was also included, a composite score of both parents’ education levels and household wealth based on principal component analysis. Household wealth in K2 was measured by the ownership of a set of household assets, while in Grade 2, it was indicated by the household income level. We also included the region of the family residence, which was a variable of four categories: urban Shanghai (reference group), suburban Shanghai, and urban Guizhou, rural Guizhou.

## Analytic Strategies

We first examined the EAP-ECDS (SF) test-retest reliability over K2 and K3. Next, we investigated the association between EAP-ECDS (SF) scores measured in K2 with school readiness assessed in K3 and academic achievement in Grade 2.

While the Pearson *r* coefficient is a standard index of test-retest reliability, it does not provide any insight into systematic errors that may be inherent in the measurement obtained with a specific tool [25]. Therefore, in addition to the Pearson *r* coefficient, we computed intraclass correlation coefficients (ICCs), which are more reliable and consider the shared method variance of identical tasks [30]. Pearson correlational values of 0.30 or less indicate low correlations, values between 0.31 and 0.60 indicate moderate correlations, and values of 0.61 and higher indicate high correlations [31]. Concerning ICC, values less than 0.50 indicate poor reliability, values between 0.5 and 0.75 indicate moderate reliability, and values of 0.75 or higher indicate good reliability [32]. Estimates of ICCs and confidence intervals should be considered to determine whether a measurement tool is reliable [32].

Following Koo and Li’s (2016) guidance and Aldridge et al.‘s (2017) recommendation, ICC estimates and their 95% confidence intervals were calculated based on an absolute, one-way random-effects model. The analysis was replicated for the total EAP-ECDS (SF) measure and its subscales, with age-specific standardized scores. As children were nested within classrooms and kindergartens, partial correlations were also conducted by running three-level linear models, using child age, gender, length of exposure to preschool education, family SES, region of household residence, and home learning environments as covariates.

The examination of predictive validity began with computing Pearson *r* statistics between K2 EAP-ECDS (SF), K3 school readiness, and Grade 2 academic achievements. Then, to evaluate the predictive power of the EAP-ECDS on school readiness at K3, Grade 2 language and literacy, and Grade 2 mathematic scores, the three latter outcome variables were regressed onto the K2 EAP-ECDS (SF) and each of its subscales, respectively. We used three-level linear models, with children representing the level-1 units, the classroom representing the level-2 units, and kindergarten representing the level-3 units. The ICC values at both the classroom and kindergarten levels ranged from 0.36 to 0.49 for the outcome variables. The models controlled for child age, gender, length of child’s exposure to preschool education, family SES, region of household residence, and home learning environments. All control variables were identified to be important for child development. In the regression models, all continuous outcome variables and predictors were standardized. Considering the pattern of missing values, the high proportion was primarily because we conducted the Grade 2 tests during the Covid-19 outbreak. It became more difficult to follow the participants than usual. We used multiple imputations with chained equations to deal with the missing values, as they could predict missing values based on our rich data set [33]. The imputation generated 25 datasets for the analysis. It was indicated that results from the analyses with the imputed dataset were similar to that from listwise deletion.

## Results

### Examining Test-Retest Reliability of the EAP-ECDS (SF)

Table 2 presents raw scores on the EAP-ECDS (SF) obtained at K2 and K3 and Pearson *r* statistics and ICCs indicating correlations between the scores obtained from the two time points. Children scored significantly higher on the total EAP-ECDS (SF) and all its seven subscales in K3 than in K2 (*p*s < 0.001). EAP-ECDS (SF) scores obtained in K2 were significantly correlated with EAP-ECDS (SF) scores in K3 (*r* = .58, p < .001). Subscale scores obtained in K2 were also significantly associated with those obtained in K3 (*r*s = 0.15–0.58, *p*s < 0.001), except for the Health, Hygiene, and Safety domain. The Cognitive Development domain (*r* = .58) and Language and Emergent Literacy domain (*r* = .42) had the highest correlations between the corresponding scores obtained at two time points.

**Table 2:** Correlation, ICC, and 95% confidence interval statistics for test-retest reliability analysis

	K2 raw score M (SD)	K3 raw score M (SD)	Pearson *r*	ICC	95% CI
EAP-ECDS (SF)	70.55 (13.73)	90.61 (7.87)	0.58***	0.73	0.68–0.77
Cognitive Development	17.58 (5.62)	24.39 (3.46)	0.58***	0.74	0.68–0.78
Social-emotional Development	12.77 (3.49)	14.9 (2.56)	0.24***	0.39	0.27–0.48
Motor Development	4.7 (1.47)	6.48 (0.94)	0.15***	0.26	0.13–0.38
Language and Emergent Literacy	14.14 (4.14)	19.81 (2.17)	0.42***	0.59	0.52–0.66
Health, Hygiene, and Safety	10.13 (1.41)	10.67 (0.74)	0.07	0.12	-0.05-0.26
Cultural Knowledge and Participation	6.36 (2.3)	8.58 (2.2)	0.21***	0.35	0.23–0.45
Approaches to Learning	4.9 (1.67)	5.79 (0.92)	0.15***	0.26	0.12–0.38

The total EAP-ECDS (SF) demonstrated good test-retest reliability, as indicated by an ICC of 0.73 (95% *CI*: 0.68–0.77). Test-retest reliability varied across subscales. Cognitive Development had the highest reliability (*ICC* = 0.74, 95% *CI* = 0.68–0.78), followed by Language and Emergent Literacy domain (*ICC* = 0.59, 95% *CI* = 0.52–0.66). These two subscales had good reliability. Test-retest reliability for other subscales (i.e., Moral Development, Health, Hygiene, and Safety, Health, Hygiene, and Safety, and Approaches to Learning) seemed unsatisfactory, as indicated by *ICC*s values below 0.50.

Table 3 shows the partial correlation between EAP-ECDS (SF) scores and its subscales obtained in K2 and K3. The first model used the K3 EAP-ECDS (SF) score as the outcome variable and K2 EAP-ECDS (SF) score as the main predictor, controlling for child age, gender, the dosage of preschool experience, home learning environments, family SES, and region of residence. This model was replicated seven times for each of the seven EAP-ECDS (SF) subscales, with only one subscale score entered each time. After adjusting for child and family background characteristics, the overall score of EAP-ECDS (SF) in K2 significantly predicted the overall EAP-ECDS (SF) in K3 (β = 0.34, *p* < .001). All the EAP-ECDS (SF) subscales obtained in K2 significantly predicted the retest subscale scores in K3, except for the Health, Hygiene, and Safety domain. Cognitive Development in K2 had the most substantial prediction of Cognitive Development in K3 (β = 0.36, *p* < .001), followed by Language and Emergent Literacy and Social-emotional Development (βs = 0.23, *p*s < 0.001). These results replicated the Pearson correlational analyses mentioned above.

**Table 3:** Predicting K3 EAP-ECDS (SF) from K2 EAP-ECDS (SF) scores

	K3 EAP-ECDS (SF)	
Predictors	β	*SE*
K2 EAP-ECDS (SF)	0.34***	0.05
K2 EAP-ECDS (SF) Subscales		
EAP-ECDS (SF): Cognitive Development	0.36***	0.08
EAP-ECDS (SF): Social-emotional Development	0.23***	0.05
EAP-ECDS (SF): Motor Development	0.16**	0.05
EAP-ECDS (SF): Language and Emergent Literacy	0.23***	0.04
EAP-ECDS (SF): Health, Hygiene, and Safety	0.06	0.05
EAP-ECDS (SF): Cultural Knowledge and Participation	0.16**	0.04
EAP-ECDS (SF): Approaches to Learning	0.17***	0.05
Child age in months	0.005	0.04
Child gender	-0.05	0.06
Child’s preschool exposure	-0.01	0.05
Family SES	0.09	0.06
Home learning environments	0.03	0.04
Suburban Shanghai residence	-0.29	0.17
Urban Guizhou	-0.88***	0.22
Rural Guizhou	-1.22***	0.25

*Note.* Estimates for covariates were generated from the model with the total EAP-ECDS (SF) score

*p < .05. **p < .01. ***p < .001

### Predictive Validity Analyses

#### Correlations Between EAP-ECDS (SF) Scores Obtained in K2 and Child Outcomes Obtained in K3 and Grade 2

The correlations between EAP-ECDS (SF) assessed in K2 and child outcomes obtained in K3 and Grade 2 are shown in Table 4. The overall score of the EAP-ECDS (SF) in K2 was significantly correlated with school readiness obtained in K3 (*r* = .59), language and literacy obtained in Grade 2 (*r* = .43), and mathematics performance assessed in Grade 2 (*r* = .50). School readiness in K3 (*r*s = 0.12–0.61) was significantly correlated with all the subscale EAP-ECDS (SF) scores obtained in K2. Grade 2 language and literacy (*r*s = 0.18–0.43) and mathematics performance (*r*s = 0.2–0.55) assessed in Grade 2 was correlated with all subscales of EAP-ECDS (SF) obtained in K2, except for the latter with Motor development.

**Table 4:** Correlations among key outcome variables

	1	2	3	4	5	6	7	8	9	10	11
**1. EAP-ECDS SF (K2)**											
2. Cognitive Development	0.86***										
3. Social-emotional Development	0.67***	0.37***									
4. Motor Development	0.33***	0.21***	0.15***								
5. Language and Emergent Literacy	0.79***	0.63***	0.37***	0.19***							
6. Health, Hygiene, and Safety	0.47***	0.31***	0.42***	0.09*	0.21***						
7. Cultural Knowledge and Participation	0.57***	0.36***	0.40***	0.11**	0.30***	0.24***					
8. Approaches to Learning	0.62***	0.51***	0.31***	0.14***	0.44***	0.22***	0.31***				
**9. EAP-ECDS SF (K3)**	0.68***	0.66***	0.39***	0.17***	0.56***	0.21***	0.31***	0.45***			
**10. School readiness (K3)**	0.59***	0.62***	0.26***	0.12**	0.46***	0.20***	0.25***	0.43***	0.61***		
**11. Language and literacy (Grade 2)**	0.43***	0.43***	0.18**	0.15**	0.36***	0.16**	0.25***	0.32***	0.35***	0.42***	
**12. Mathematics (Grade 2)**	0.50***	0.55***	0.20***	0.11	0.39***	0.21***	0.25***	0.32***	0.37***	0.45***	0.76***

*Note.* *p < .05. **p < .01. ***p < .001

#### Results From the Regression Models

Table 5 shows the results from the three-level linear model examining the prediction of EAP-ECDS (SF) assessed in K2 on school readiness assessed in K3. After controlling for child and family covariates, the overall score of EAP-ECDS (SF) in K2 was significantly associated with school readiness (β = 0.26, *p* < .001), indicating a moderate prediction effect of EAP-ECDS (SF) on later school readiness. The three-level model was replicated seven times for each of the seven EAP-ECDS (SF) subscales. Except for Motor development, all K2 EAP-ECDS (SF) subscales predicted K3 school readiness significantly, with the strongest predictors being Cognitive Development (β = 0.23, *p* < .001) and Approaches to Learning (β = 0.17, *p* < .001).

**Table 5:** Predicting K3 school readiness from K2 EAP-ECDS (SF) scores

	School readiness	
Predictors	β	SE
K2 EAP-ECDS (SF)	0.26***	0.05
K2 EAP-ECDS (SF): Cognitive Development	0.23***	0.06
K2 EAP-ECDS (SF): Social-emotional Development	0.13***	0.04
K2 EAP-ECDS (SF): Motor Development	0.02	0.03
K2 EAP-ECDS (SF): Language and Emergent Literacy	0.13**	0.04
K2 EAP-ECDS (SF): Health, Hygiene, and Safety	0.09*	0.05
K2 EAP-ECDS (SF): Cultural Knowledge and Participation	0.11*	0.04
K2 EAP-ECDS (SF): Approaches to Learning	0.17***	0.04
Child age in months	0.05	0.04
Child gender	0.04	0.06
Child’s preschool exposure	-0.03	0.04
Family SES	0.07	0.07
Home learning environments	-0.004	0.04
Suburban Shanghai residence	-0.003	0.09
Urban Guizhou	-0.97***	0.16
Rural Guizhou	-1.57***	0.17

*Note.* Estimates for covariates were generated from the model with the total EAP-ECDS (SF) score

*p < .05. **p < .01. ***p < .001

Table 6 shows the results from the three-level linear model examining the prediction of K2 EAP-ECDS (SF) on Grade 2 academic achievements. Again, we first conducted the three-level model with the overall K2 EAP-ECDS (SF), followed by models with each of the seven EAP-ECDS (SF) subscale scores. After controlling for the important child- and family-related characteristics, the overall score of K2 EAP-ECDS (SF) was significantly associated with Grade 2 language and literacy skills (β = 0.18, *p* < .001) and mathematics performance (β = 0.22, *p* < .001), indicating a moderate size of prediction effect of EAP-ECDS (SF) on later academic achievements. Cognitive Development, Language and Emergent Literacy, and Approaches to Learning were significant predictors of language and literacy skills, albeit with moderate effect sizes. Only the Cognitive Development and Language and Emergent Literacy domains were significant predictors of mathematics skills.

**Table 6:** Predicting Grade 2 academic outcomes on K2 EAP-ECDS (SF) scores

	Language and literacy			Mathematics	
Predictors	β	SE		β	SE
K2 EAP-ECDS (SF)	0.18*	0.07		0.22**	0.07
K2 EAP-ECDS (SF): Cognitive Development	0.17*	0.09		0.20*	0.08
K2 EAP-ECDS (SF): Social-emotional Development	-0.002	0.06		0.1	0.06
K2 EAP-ECDS (SF): Motor Development	0.01	0.06		0.04	0.05
K2 EAP-ECDS (SF): Language and Emergent Literacy	0.17**	0.06		0.15*	0.06
K2 EAP-ECDS (SF): Health, Hygiene, and Safety	0.03	0.06		0.09	0.06
K2 EAP-ECDS (SF): Cultural Knowledge and Participation	0.09	0.05		0.09	0.05
K2 EAP-ECDS (SF): Approaches to Learning	0.13*	0.06		0.04	0.06
Child age in months	0.07	0.08		0.09	0.06
Child gender	-0.19	0.12		-0.03	0.09
Child’s preschool exposure	-0.09	0.08		-0.02	0.07
Family SES	0.02	0.07		0.02	0.07
Home learning environments	0.07	0.06		-0.004	0.06
Suburban Shanghai residence	0.07	0.18		-0.09	0.2
Urban Guizhou	-0.45	0.27		-0.85*	0.25
Rural Guizhou	-1.13	0.6		-1.01	0.56

*Note.* Estimates for covariates were generated from the model with the total K2 EAP-ECDS (SF) score

*p < .05. **p < .01. ***p < .001

## Discussion

Direct assessment of early child development has been considered an effective method to generate quality data on early child development. Valid and reliable measurement tools are necessary to guide decisions on programs and policies. This study examined the test-retest reliability of the EAP-ECDS (SF) by evaluating the degree of association between the EAP-ECDS (SF) obtained in K2 and the score obtained at the end of K3. It also examined the predictive validity of the scale by examining the association between EAP-ECDS (SF) score obtained in K2 and children’s school readiness assessed in K3 and children’s academic performance in Grade 2. This is the first study that examined the psychometric properties of the EAP-ECDS (SF) scale using a longitudinal dataset. In general, the EAP-ECDS (SF) was shown to have good test-retest reliability and to be a good predictor of children’s subsequent academic achievement in primary schools.

### Test-Retest Reliability of the EAP-ECDS (SF)

Regarding test-retest reliability, we found that EAP-ECDS (SF) score obtained in K2 was positively and significantly correlated with EAP-ECDS (SF) score obtained in K3. Pearson correlation between the two scores was strong (*r* = .58). The EAP-ECDS (SF) in K2 predicted the retest score in K3 significantly and moderately after controlling for other background characteristics that might affect the score in K3. The *ICC* value also showed that the total EAP-ECDS (SF) was reliable. The findings suggest that the EAP-ECDS (SF) is appropriate for measuring early child development.

Meanwhile, the *ICC* values indicated that two subscales (i.e., Cognitive Development, Language and Emergent Literacy) meet the “good reliability” threshold; other subscales did not [34]. Possible explanations for the findings are complex. For example, regarding the Social-emotional Development domain, there are challenges associated with assessing young children’s social-emotional skills, given the complex constructs within this domain and the difficulty of identifying the most appropriate reporter [35]. Parents and teachers may be unable to provide adequate and accurate information on a child’s social-emotional skills, which are difficult to assess in a one-time individual assessment context. Children’s moods and relationship with the assessor may affect their performance on the scale. The scores in the Health, Hygiene, and Safety domain may relate to standard kindergarten practices regarding health and motor skills in China. Kindergartens in China emphasize health, hygiene, and safety, which may explain why scores on this subscale did not vary widely, as evidenced by small standard deviations across our sample. Motor development also did not differ much, and there have also been secular trends with decreasing motor skills among young children in China [22]. Motor skills may have also been affected by children’s overall activity levels when data were collected. K2 data were collected in winter when children’s outdoor physical activities were limited because of the cold weather in the research sites. Still, the retest was administrated in summer when children were provided with more opportunities for outdoor physical activities. Further, although *ICC* has been considered a valuable index for test-retest reliability, there are no standard values for acceptable test-retest reliability using *ICC* [32]. Research on absolute test-retest reliability is necessary to generate additional evidence on the reliability of the total scale and subscales.

### Predictive Validity of the EAP-ECDS (SF)

The knowledge, skills, and capacities that children gain during their preschool years lay a strong foundation for their future learning and academic success. Extensive evidence has shown that early childhood developmental outcomes predict later academic achievement [36–38]. Therefore, a good early development measurement tool is expected to predict children’s subsequent performance at school. Regarding predictive validity, we found moderate and significant correlations between the EAP-ECDS (SF) score assessed in K2 and school readiness assessed in K3 and between K2 EAP-ECDS (SF) and academic achievement in Grade 2. The significant association between EAP-ECDS (SF) score assessed in K2 and child outcomes assessed in K3, and Grade 2 remained after adjusting for a series of child and family background covariates. The findings suggested that the EAP-ECDS (SF) predicted children’s school readiness and future academic performance. Further, the results indicated that among the seven EAP-ECDS (SF) domains, Cognitive Development and Language and Emergent Literacy seemed to have higher reliability and validity than other domains. In the meantime, after considering background characteristics, the small coefficients indicated that children who lag behind their peers during preschool would likely catch up in primary schools.

### Recommendations for Practice

The EAP-ECDS (SF) was initially developed to reflect young children’s holistic development, and the domains assessed were related to each other [11]. Based on the study’s findings, we recommend that the total EAP-ECDS (SF) be used when evaluating the holistic development of preschool-aged children because of its higher reliability, validity, and interrelations across different domains. Three cognition-related domains – Cognitive Development, Language and Emergent Literacy, and Approaches to Learning – could be selected to predict future academic success if resources do not permit the administration of the full version of the EAP-ECDS (SF).

## Limitations

Strengths of this study include the diverse family background of the sample and longitudinal predictive validity of the EAP-ECDS (SF) by assessing children as they progress to primary grades. Nonetheless, it is important to note limitations. The long interval and child age in K3 may adversely influence test-retest reliability. Some children assessed in K3 were slightly older than 5 years, while the EAP-ECDS (SF) was designed to evaluate the development of 3- to 5-year-olds. In addition, environmental conditions during the data collection period may have affected children’s performance on the measure. For example, children’s opportunities for outdoor activities may differ in winter (data collection period for K2) and summer (data collection period for K3), which may have affected children’s performance on motor development measures.

Further research utilizing EAP-ECDS (SF) or similar early child development measures is recommended to investigate test-retest reliability by collecting data within a shorter interval and focusing more on children under six years. Lastly, due to school suspensions and restrictions associated the COVID-19 pandemic, we were not able to conduct the Grade 2 academic assessments in classrooms. Instead, the tests were sent to the children’s home and children completed the tests under the supervision of a parent. Children may have experienced more distractions at home that they would have in a classroom setting. Their competence may also be underestimated as there was no teacher supervision and children may be more compliant to teachers’ requests to complete the tests than to parents’ requests to do so.

## Summary

The East Asia-Pacific Early Child Development Scales (EAP-ECDS) Short Form was developed to assess the development of 3- to 5-year-olds. This study examined the reliability and predictive validity of the Scales among a sample of children in rural and urban China. Children who attended kindergarten were administered the EAP-ECDS Short Form at ages 4 and 5. Their school readiness was assessed before they started primary school, and they completed literacy and mathematics achievement tests in Grade 2. Results confirmed that the EAP-ECDS has good psychometric properties. Test-retest reliability was high, and EAP-ECDS Short Form scores at age 4 predicted school readiness and achievement in Grade 2. The Scales may be used for population-level assessment and programme evaluation in East Asia.
